# Thrombotic thrombocytopenic purpura following ChAdOx1 nCov-19 vaccination: A case report

**DOI:** 10.1016/j.idcr.2023.e01795

**Published:** 2023-05-08

**Authors:** Bahareh Shateri Amiri, Hanieh Radkhah, Reza Taslimi, Zahra Shahbazi Dastjerdi, Mohamad Mehdi Khadembashiri, Milad Gholizadeh Mesgarha, Shiva Rahimipour Anaraki

**Affiliations:** a Department of Internal Medicine, School of Medicine, Hazrat-e Rasool General Hospital, Iran University of Medical Sciences, Islamic Republic of Iran; b Department of Internal Medicine, School of Medicine, Sina Hospital, Tehran University of Medical Sciences, Tehran, Islamic Republic of Iran; c Department of Internal Medicine, Imam Khomeini Hospital, Tehran University of Medical Sciences, Tehran, Islamic Republic of Iran; d Students’ Scientific Research Center, Tehran University of Medical Sciences, Tehran, Islamic Republic of Iran; e Faculty of Medicine, Iran University of Medical Sciences (IUMS), Tehran, Islamic Republic of Iran

**Keywords:** SARS-CoV-2, Vaccine, Microangiopathic hemolytic anemia, Thrombotic microangiopathy, AstraZeneca, AZD1222

## Abstract

Vaccine-associated thrombotic thrombocytopenic purpura (TTP) is a rare type of acquired TTP recently reported after COVID-19 vaccination. Merely four cases are ascribed to the ChAdOx1 nCoV-19 vaccine in the medical literature till the preparation of this study. In this case report, we describe a 43-year-old man who developed symptoms of TTP four days after receiving the second dose of the ChAdOx1 nCoV-19 vaccine. Peripheral blood smear demonstrated multiple schistocytes. Given a high plasmic score, he received plasma exchange, corticosteroids, and rituximab, and later, low ADAMTS 13 activity and high-titer ADAMTS inhibition antibody confirmed the diagnosis of COVID-19 vaccine-associated TTP. COVID-19 vaccine-associated TTP is an infrequent consequence of SARS-CoV-2 vaccination but with a substantial mortality rate which must be considered as one of the crucial differential diagnoses of post-COVID-19 vaccine thrombocytopenia besides vaccine-induced immune thrombotic thrombocytopenia and Immune thrombocytopenic purpura.

## Introduction

ADAMTS13 deficiency–mediated thrombotic microangiopathy, commonly known as Thrombotic Thrombocytopenic Purpura (TTP), is a rare but fatal condition insofar as its mortality approaches high as 90 % without therapeutic intervention [1]. This hematologic emergency is classified based on hereditary or acquired ADAMTS13 deficiency; the latter, the more common form, is caused by ADAMTS13 autoantibodies [2].

Vaccine-associated TTP is a rare type of acquired TTP reported following the administration of various vaccines comprising Influenza Seasonal, Pneumococcal, Triple Diphtheria-Tetanus Poliomyelitis, Rabies, and recently COVID-19 vaccines that express high ADAMTS13 autoantibodies titer in most of the reported cases [3], [4].

COVID-19 vaccine-associated TTP is an entity limited to a few case reports and small case series [5]. Herein, we introduce a case of de novo TTP, which occurred after receiving the second dose of the adenoviral vector vaccine, and then briefly discuss and review the current medical literature on this critical adverse event of COVID-19 vaccines.

## Case report

A 43-year-old man was admitted to our hospital with a complaint of fever and icterus 15 days after the COVID-19 vaccination. He reported that his symptoms had started with epigastric pain and nausea four days after vaccination with the second dose of the ChAdOx1 nCoV-19 vaccine. After that, he developed diffuse pruritic rashes, which abated with chlorpheniramine. One day later, he was admitted to a hospital with a complaint of dyspnea and fever. Evaluation with chest CT with and without contrast was negative for any notable pathologic findings such as COVID-19 infection or pulmonary embolism. COVID-19 PCR showed a negative result, and His laboratory data were normal during this admission. He was discharged within four days after receiving oral antibiotics and supportive treatment. Two days after discharge, his new symptoms began with severe arthralgia accompanied by severe weakness and continuous fever, and in the next five days, icterus, dark urine, and drowsiness were added to his symptoms.

The patient's past medical and family history was unremarkable for any thrombotic events or autoimmune diseases. He received his first dose of COVID-19 vaccination of the same vaccine type ten weeks before his second dose and only had a transient fever and myalgia for two days. On physical examination, he was drowsy but oriented and answered relevant questions. Oxygen saturation was 90 % when he breathed in ambient air and 98 % with nasal oxygen. His temperature was 38.7 °C, pulse rate was 100 beats per minute, and blood pressure was 110/70 mmHg. His sclera and skin were icteric, and tenderness was noted over his wrists and small joints of upper and lower extremities, but no signs of erythema, warmth, or swelling were observed. Examination of the lung, abdomen, and nervous system was otherwise normal.

On the third day of admission, he developed a tonic-clonic seizure for 3 min that terminated with midazolam. After the post-ictal phase, the patient was conscious without any focal neurological deficit, but after about one hour, he became tachypneic. Accordingly, he was admitted to the intensive care unit, and on the next day, he was intubated because of status epilepticus. His brain CT scan and chest radiography were normal at that time. His new lab data showed progressive thrombocytopenia, anemia, increased creatinine, and high LDH (lactate dehydrogenase) in favor of hemolytic anemia. Laboratory test results during the hospital stay are shown in Table 1.

**Table 1:** The course of the patient's lab tests during admission.

Test	Admission day	Day 3	Day 8	Discharge	Reference range
White blood cell count (× 10^9^/L)	7.4	6.7	8.6	5.5	4–11
Hemoglobin level (g/dL)	14.4	9.6	8.8	10.2	12.5–17.5
Platelet count (× 10^9^/L)	130	85	45	167	150–400
MCV (Femtoliter)	75	72	68		80–100
CRP (mg/dL)	70		76		< 0.3
Creatinine (mg/dL)	1.5	1.7	1.9	1.1	0.7–1.3
AST (U/L)	96	251		46	10–35
ALT (U/L)	106	98		34	10–35
ALP (IU/L)	256				45–145
Indirect Bilirubin (mg/dL)	4.4			1.6	< 0.3
Total Bilirubin (mg/dL)	6.8			2.5	0.1–1.2
LDH (U/L)	2196	2366	2246	532	< 300

**Fig. 1:**
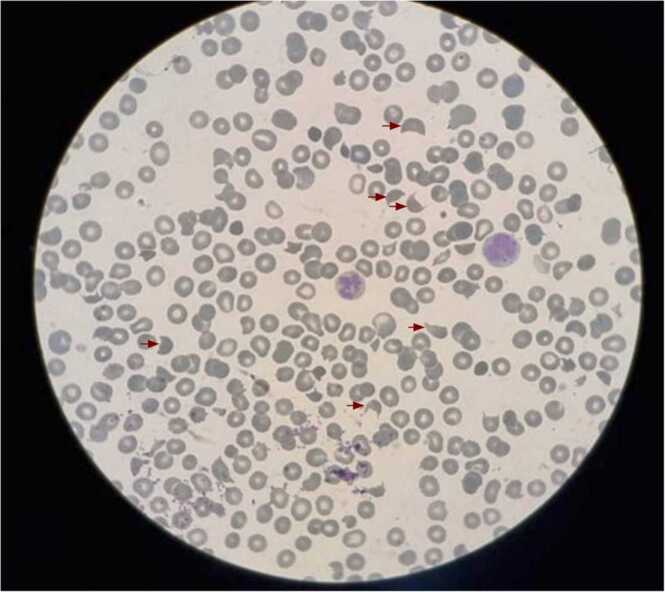
Peripheral blood smear depicts abundant schistocytes (6 %) (red arrows) due to microangiopathic hemolytic anemia.

Peripheral blood smear of the patient revealed abundant schistocytes (6 %) in favor of microangiopathic hemolytic anemia diagnosis (Fig. 1).

According to the patient's high plasmic score Daily, plasmapheresis was performed, and dexamethasone was administered immediately. Specific diagnostic lab tests for TTP and rheumatologic diseases were sent. Despite five days of treatment with plasmapheresis, platelet count did not increase appropriately; therefore, plasmapheresis was scheduled to be done twice daily, and rituximab was also added to the treatment.

His lab data showed a significant reduction in ADAMTS 13 activity (< 10 %, normal reference range: 50–160 %) and a high level of ADAMTS 13 inhibition antibody (16 U/mL, normal reference range: < 12 U/mL), which confirmed the diagnosis of TTP. Evaluation for possible underlying rheumatologic diseases and HIV tests were negative. His plasmapheresis was discontinued when his platelet rose to the normal count range and his LDH level diminished (Table 1). Moreover, the patient was extubated as he regained his normal level of consciousness. Eventually, the patient was discharged with weekly rituximab for four weeks, and in his follow-up, he was doing well without recurrence of his symptoms.

## Discussion

### Epidemiology

Various autoimmune platelet disorders, such as vaccine-induced immune thrombotic thrombocytopenia (VITT), immune thrombocytopenic purpura (ITP), and TTP have been reported following the administration of COVID-19 vaccination [6], [7], [8], [9]. Compared to COVID-19 vaccine-associated ITP, COVID-19 vaccine-associated TTP is less commonly reported but leads to more mortality rate. In a systematic review by Bidari et al. among 77 patients of COVID-19 vaccine-associated ITP, only one death was reported (1.3 %) but considering COVID-19 vaccine-associated TTP, we have found 40 cases with three fatality cases (7.5 %) based on a comprehensive review of the literature [5], [7], [10], [11].

In comparison with TTP due to other causes than the COVID-19 vaccine, it seems the COVID-19 vaccine-associated TTP has a less fatal course; with timely appropriate treatment, particularly plasma exchange non COVID-19 vaccine-associated TTP has a 10.3 % mortality rate but COVID-19 vaccine-associated TTP shows 7.5 % mortality rate. However, this comparably low mortality rate of COVID-19 vaccine associated TTP may ascribe to the low incidence of this adverse effect of the vaccine which is restricted to a few case reports [12].

Of these 40 cases, only four cases of COVID-19 vaccine-associated TTP have been reported following ChAdOx1 nCov-19 vaccination, and none caused fatality [5], [13]. As the fifth case of ChAdOx1 nCov-19 associated TTP, our case had a similar course with the patient’s full recovery.

### Pathophysiology

The causal relationship between acquired TTP and COVID-19 vaccination is primarily supported by temporal correlations rather than by identifying cross-reactive epitopes between antigens in these vaccines and ADAMTS13. Nonetheless, two pathophysiological mechanisms have been postulated for de-novo TTP after COVID-19 vaccination: 1) COVID-19 vaccine plays a role as a trigger to uncover an undiagnosed occult TTP to present as a full episode of the disease. It is manifested by patients with symptom onset within a few days of vaccination. 2) Autoantibodies are produced against ADAMTS13 following an immunological trigger through mechanisms of molecular mimicry [5], [14]. Both scenarios could also be possible for our case.

### Diagnosis and management

Management of COVID-19 vaccine-associated TTP should not be detained till confirmation of TTP diagnosis with low ADAMTS 13 activity, and treatment must be started with the presumptive TTP diagnosis because of its high mortality rate. Nevertheless, post-COVID-19 vaccine thrombocytopenia has another crucial differential diagnosis like VITT; therefore, ADAMTS 13 activity was checked and confirmed the diagnosis of TTP in almost all cases. Detection of low ADAMTS 13 activity also established the TTP diagnosis in our case [4], [5], [15].

Plasmapheresis and corticosteroids were the mainstays of the treatment in almost all reported cases of COVID-19 vaccine-associated TTP; a similar therapeutic strategy was employed for our patient [4], [5]. Caplacizumab, Rituximab, or a combination of them, were used for patients with virtually refractory disease courses. Caplacizumab is not available in our country, but Rituximab was added to the treatment protocol, which led to treatment response in our patient [5], [10], [16].

### Conclusion

Evaluation of post-COVID-19 vaccine thrombocytopenia is a diagnostic challenge for clinicians as serious adverse events like VITT, ITP, and TTP have been reported following SARS-CoV-2 vaccination. Scrutiny is required for taking a thorough history from patients with post-COVID-19 vaccine thrombocytopenia, educating them on warning signs and symptoms, and following them closely looking for these signs and symptoms which could harbinger of a critical ongoing condition such as COVID-19 vaccine-associated TTP.

## CRediT authorship contribution statement

B.SHA and H.R conceptualized the study and revised and prepared the final version of the manuscript. R.T and Z.SH diagnosed and treated the patient and reviewed the manuscript. MM.KH, M.GHM and SH.RA wrote the initial draft of the manuscript.

## Ethical approval

There is no ethical approval required for this descriptive report.

## Consent

No identifiable data were disclosed in this article; however, written consent was obtained from the patient and his family in this study. A copy of the written consent is available for review by the Editor-in-Chief of this journal on request.

## Funding

This study was not supported by any funding or financial support.

## Conflict of Interest

The authors declare that they have no conflict of interest.
